# The Astana Declaration and future African primary health care

**DOI:** 10.4102/phcfm.v10i1.1983

**Published:** 2018-11-28

**Authors:** Robert Mash

**Affiliations:** 1 Division of Family Medicine and Primary Care, Stellenbosch University, South Africa

At the recent Global Symposium on Health Systems,^1^ one of the participants commented that there are three trains currently running the global health agenda – the sustainable development goals, universal health coverage and primary health care. As I write this editorial, the inter-governmental meeting in Astana will be meeting to re-commit the world to primary health care as the essential and fundamental basis of cost-effective and equitable health systems.^2^ This meeting comes 40 years after the landmark Declaration of Alma Ata which rallied the world around the call of ‘health for all’ and primary health care.^3^ However, 40 years on and despite the 2008 World Health Report also declaring primary health care is needed ‘now more than ever’,^4^ the world has yet to fully realise the vision and potential of primary health care.

Nowhere is this more true than on the African continent where strong and effective primary health care is still a dream for most communities. The dilemma for architects of primary health care systems on the African continent is summed up by the observation that the African continent has 25% of the global disease burden, but only 3% of the world’s health workers and less than 1% of the world’s health expenditure.^5^

The enormous burden of disease on the African continent has also been in transition, with the rise of chronic communicable (e.g. HIV and AIDS) and non-communicable diseases (e.g. ischaemic heart disease, stroke, diabetes) alongside infectious diseases, causes of maternal and child mortality, violence and trauma.^6^

Health is just one of the key elements in the social foundation of societies and is intimately connected to others such as clean water, food security, energy, housing, income, gender equality, social equity, education, peace and political voice. The new concept of ‘doughnut economics’^7^ challenges the economy to see its function as enabling humanity to thrive within boundaries determined by the need to ensure a strong social foundation, while not exceeding the ceiling imposed by planetary environmental limits.

In Africa, however, many governments are not committed to sufficient expenditure on the health system and development of primary health care. Expenditure is often complemented by foreign aid or the activities of non-governmental and faith-based organisations which tend to focus on specific diseases and programmes. These programmes may even weaken the broader primary health care system as they create an internal brain drain into vertical programmes that have better salaries and conditions. They may also create inequity by disease as certain diseases are prioritised more than others. Governance of the health system may also be subject to corruption and irregular expenditure. As families try to access health services, they often experience catastrophic health expenditure.

The critical shortage of health workers in many countries has led to a concentration of health professionals in central and referral hospitals, while primary health care is delivered by low- or mid-level workers with limited training, poor infrastructure, few resources and inadequate supportive supervision. Health professionals are difficult to retain and may also migrate to opportunities in high-income countries such as the United Kingdom (UK), United States (US), Canada and Australia.

In this context, it is not surprising that primary care fails to deliver on its core characteristics of accessibility, comprehensiveness, continuity, coordination of care, person-centredness and quality.^8^

Despite this rather gloomy picture, the Astana Declaration should once again open up a window of opportunity for countries to focus on building primary health care as the foundation of their health systems rather than its last neglected outpost. In the African context, I believe there is a number of ways in which this can be done.

Universal health coverage requires strong primary health care, and in several countries national health insurance is making a package of primary health care services available to all with no user fees when accessing the service. Ghana has established such a scheme and South Africa is in the process of doing so.

Community-orientated primary care is:

a continuous process by which primary health care is provided to a defined community on the basis of its assessed health needs, by the planned integration of primary care practice and public health.^9^

In Africa this is usually dependent on community health workers working in a multidisciplinary primary health care team for a defined community. The approach provides access to primary care and assesses the health needs of the community in a participatory approach that leads to appropriate interventions.

Family medicine is emerging and becoming established in many countries; it is defined as the subset of services offered by doctors trained as specialists in family medicine within the district health system.^10^ These family physicians bring much needed generalist expertise into district hospitals and primary health care teams and function as consultants, capacity builders and leaders of clinical governance to improve the quality of care in the whole team.

Evaluation of primary health care requires an appropriate framework such as the one offered by the Primary Health Care Performance Initiative,^11^ and appropriate tools to measure key aspects such as the Primary Care Assessment Tool.^12^ In the absence of ‘big data’ and electronic records, we have to collect routine and periodic data to measure the strength of primary health care. Such data should lead to generic and targeted interventions designed to improve primary health care performance and clinical processes. There is also a need for more primary care research to inform policymakers on the best interventions in our context.^13^

Despite the failures of the previous 40 years to deliver on the vision of the Alma Ata Declaration, the new Astana Declaration should create an opportunity for countries to re-commit to primary health care. A number of initiatives already taking place in the African context point the way, such as national health insurance, community-orientated primary care, family medicine and primary health care evaluation tools.
